# Poor Appetite Negatively Affects Recovery of Swallowing Function During Post‐Acute Rehabilitation

**DOI:** 10.1111/ggi.70198

**Published:** 2025-09-29

**Authors:** Akio Shimizu, Xiaojing Sharon Wu, Shinsuke Nagami, Katsuya Nakamura, Jun Kayashita, Akiko Nomoto, Ichiro Fujishima, Ryo Momosaki

**Affiliations:** ^1^ Department of Rehabilitation Medicine Mie University Graduate School of Medicine Tsu City Mie Japan; ^2^ School of Population Health, Faculty of Medical and Health Sciences University of Auckland Auckland New Zealand; ^3^ Department of Communication Disorders, School of Rehabilitation Sciences Health Sciences University of Hokkaido Ishikari‐gun Hokkaido Japan; ^4^ Department of Speech‐Language Pathology and Audiology, Faculty of Rehabilitation Kawasaki University of Medical Welfare Kurashiki City Okayama Japan; ^5^ Department of Health Sciences, Faculty of Human Culture and Science Prefectural University of Hiroshima Hiroshima City Hiroshima Japan; ^6^ Department of Dentistry Hamamatsu City Rehabilitation Hospital Hamamatsu Shizuoka Japan; ^7^ Department of Rehabilitation Medicine Hamamatsu City Rehabilitation Hospital Hamamatsu Shizuoka Japan

**Keywords:** appetite, dysphagia, nutritional assessment, older adults, rehabilitation, swallowing function

## Abstract

**Purpose:**

Impaired swallowing function is prevalent among older adults, significantly impacting nutritional status and quality of life. Although appetite plays a crucial role in nutrition and clinical recovery, its influence on swallowing function recovery remains unclear. This study aimed to clarify the relationship between poor appetite and swallowing recovery in older adults undergoing rehabilitation.

**Methods:**

This retrospective cohort study analyzed data from 309 hospitalized older adults (aged ≥ 65 years) admitted to a post‐acute rehabilitation ward with diagnosed impaired swallowing function. Appetite at admission was assessed using the Simplified Nutritional Appetite Questionnaire for Japanese Elderly (SNAQ‐JE). Swallowing function was evaluated at discharge using the Food Intake Level Scale (FILS). Multivariable analyses adjusted for age, sex, nutritional status, sarcopenia, cognitive function, functional independence, and clinical characteristics.

**Results:**

Among participants (mean age 80.3 ± 7.9 years; 57.6% female), 41.4% had poor appetite. Patients with poor appetite showed significantly lower FILS scores at discharge compared to those with normal appetite (*p* = 0.002). In adjusted analyses, poor appetite remained negatively associated with FILS scores (Estimate: −0.224, 95% CI: −0.435 to −0.013, *p* = 0.038). Similarly, poor appetite was associated with impaired swallowing function at discharge (odds ratio: 2.11, 95% confidence interval: 1.16–3.92, *p* = 0.016).

**Conclusions:**

Poor appetite was negatively associated with the recovery of swallowing function among older patients undergoing post‐acute rehabilitation. Early evaluation of appetite may assist in identifying barriers to the recovery of swallowing function within this population.

## 1 Introduction

Impaired swallowing function is a common complication in older adults, often caused by various age‐related conditions such as stroke, dementia, neurological disorders, and sarcopenia [1]. Among hospitalized older patients, impaired swallowing function represents a clinically significant issue due to its strong associations with malnutrition, aspiration pneumonia, and increased mortality risk [2]. Furthermore, impaired swallowing function often limits oral appetite, exacerbating nutritional deficits [3]. Older patients with impaired swallowing function are considered a vulnerable population, facing these interrelated health issues. Therefore, clinical management of older patients with swallowing dysfunction requires early identification of poor appetite and malnutrition, alongside timely and targeted nutritional interventions.

Poor appetite is another prevalent issue in older adults. Previous studies have demonstrated that reduced appetite increases vulnerability in older adults by contributing to conditions such as sarcopenia [4] and frailty [5]. Additionally, poor appetite has been shown to be a factor hindering improvements in activities of daily living (ADL) among hospitalized patients with stroke [6]. Inadequate nutrient intake likely mediates these effects. Therefore, enhancing appetite among hospitalized older patients might play a crucial role in optimizing their overall functional recovery.

Poor appetite may negatively affect recovery of swallowing function in hospitalized older patients with impaired swallowing function. Poor appetite and impaired swallowing function are closely related and both conditions adversely impact oral food intake. A previous study suggests that adequate energy intake may be beneficial for the recovery of swallowing function [7]. However, the association between poor appetite and recovery of swallowing function has not been clearly established. Clarifying this association could further emphasize the importance of appetite‐focused interventions for hospitalized older patients with impaired swallowing function. Therefore, this study aimed to examine whether poor appetite negatively affects recovery of swallowing function in hospitalized older adults with impaired swallowing function.

## 2 Methods

### 2.1 Study Design and Participants

This retrospective cohort study was conducted using medical records from Hamamatsu City Rehabilitation Hospital. Participants included patients aged 65 years or older, admitted between January 2019 and December 2021 for comprehensive rehabilitation therapy. In the Japanese healthcare system, comprehensive inpatient rehabilitation is typically provided for three major categories of conditions: cerebrovascular disease (primarily stroke), musculoskeletal disorders (including fractures and joint diseases), and hospital‐associated deconditioning. In this study, patients received swallowing function rehabilitation alongside physical and occupational therapy, based on clinical judgment by their primary physicians. Eligibility criteria included the presence of impaired swallowing function at admission. Based on previous research, impaired swallowing function was defined as a Food Intake Level Scale (FILS) [8] level of 8 or below at admission [7]. FILS level 8 is defined as “the patient eats three meals daily but excludes foods that are particularly difficult to swallow.” [8]. Exclusion criteria included patients who were transferred to another hospital due to deterioration of their medical condition during hospitalization, as well as those who declined or were unable to complete the appetite questionnaire, and patients whose oral intake was classified as FILS level 6 or lower, as this category includes patients with severe dysphagia. This study received ethical approval from the Ethics Committee of Hamamatsu City Rehabilitation Hospital (approval ID: 19–77). Due to the retrospective nature, obtaining written informed consent from each participant was not feasible. Therefore, an opt‐out approach was adopted, whereby participants were notified of the study and provided an opportunity to decline participation through announcements placed on the hospital's official website and notice boards.

### 2.2 Appetite Assessment

Poor appetite was assessed using the Simplified Nutritional Appetite Questionnaire for Japanese Elderly (SNAQ‐JE) [9], which has been previously validated for reliability and validity in older Japanese adults. The SNAQ‐JE comprises four items evaluating appetite, satiety, taste perception, and general mood, with each item rated on a 5‐point scale ranging from 1 to 5. Based on a previous study, participants with a total SNAQ‐JE score of 14 or lower were classified as having poor appetite [9].

### 2.3 Outcome Measurements

The primary outcome of this study was the recovery of swallowing function during hospitalization, assessed using the FILS. The FILS is a validated clinical tool designed to objectively measure dysphagia severity based on patients' oral intake capacity [8]. The FILS consists of 10 levels, ranging from level 1 (complete inability to ingest any food orally) to level 10 (normal oral intake without dietary restrictions). FILS assessments were conducted at both admission and discharge, allowing quantification of changes in swallowing function over the course of rehabilitation. Higher FILS levels indicated greater swallowing function and increased oral intake capacity. By comparing FILS scores at admission and discharge, we assessed the extent of clinical changes in swallowing function during their hospital stay. Additionally, if a significant association between poor appetite and swallowing function changes was observed, further analyses were performed to identify specific components of poor appetite associated with this outcome.

The secondary outcome was the presence of impaired swallowing function at discharge, defined as a FILS level of 8 or lower, serving as a proxy indicator for insufficient functional recovery.

### 2.4 Other Variables

Demographic variables included patient age at admission (in years) and sex. Clinical variables included the primary diagnosis necessitating rehabilitation therapy, categorized as stroke, musculoskeletal disorders, or hospital‐associated deconditioning. Additionally, the time interval (in days) between the onset of the condition and admission to the rehabilitation facility was recorded.

Comorbidities were evaluated using the Charlson Comorbidity Index (CCI), a widely used metric quantifying the overall burden of chronic diseases [10]. Nutritional status was assessed using the Malnutrition Universal Screening Tool (MUST), a validated instrument to detect malnutrition risk based on body mass index, recent body weight loss, and acute illness severity [11]. Skeletal muscle mass was measured using the skeletal muscle mass index (SMI) via bioelectrical impedance analysis (Inbody S10; Inbody, Tokyo, Japan), while muscle strength was evaluated using handgrip strength (HGS) with a digital dynamometer (MG‐4800; CHARDER Electronic, Taichung, Taiwan). Sarcopenia was diagnosed according to the Asian Working Group for Sarcopenia 2019 criteria [12], defined as the presence of both low muscle mass (SMI) and low muscle strength (HGS). Functional independence at baseline was assessed using the Functional Independence Measure (FIM) [13], a validated instrument evaluating both physical and cognitive abilities required for daily living activities. FIM scores range from 18 (completely dependent) to 126 (fully independent), with higher scores indicating greater functional independence. Lastly, cognitive function was evaluated using the Mini‐Mental State Examination (MMSE) [14], a widely used and validated instrument to screen for cognitive deficits. The MMSE provides scores from 0 to 30, where lower values represent poorer cognitive performance; a score of < 24 is commonly interpreted as indicative of cognitive impairment.

### 2.5 Statistical Analysis

Continuous variables were summarized using means and standard deviations or medians and interquartile ranges, while categorical variables were reported using frequencies and percentages (%). To evaluate differences between groups stratified by appetite level, comparisons were conducted with independent *t*‐tests or Mann–Whitney U tests for continuous variables and chi‐square tests for categorical variables. Multivariable linear regression analysis was performed to examine the association between poor appetite and swallowing function at discharge, after adjusting for potential confounders. Additionally, multivariable logistic regression analysis was performed to further clarify the association between appetite status and impaired swallowing function at discharge. These multivariable regression models included the following covariates to control for their possible influence: age, sex, primary diagnosis necessitating rehabilitation, CCI, days from onset to admission, MUST score, presence of sarcopenia, FILS score at admission, FIM score at admission, and MMSE score at admission. As a sensitivity analysis, the total SNAQ‐JE score was used as a continuous variable to test the robustness of the association between appetite and swallowing function. Exploratory subgroup analyses were conducted by primary diagnosis (stroke vs. non‐stroke). The same covariate‐adjusted model was fitted within each subgroup. No interaction tests were performed, and results are presented with 95% CIs for qualitative comparison. Regression results are reported as estimates (linear) or odds ratios (logistic) with corresponding 95% confidence intervals (CI). All statistical analyses were conducted using R software version 4.2.2 (R Core Team, Vienna, Austria), and statistical significance was set at a two‐sided *p*‐value of less than 0.05.

## 3 Results

A total of 810 older patients admitted to the hospital between December 2019 and June 2021 were screened. Of these, 377 patients with normal swallowing function (FILS > 8) and 14 patients with severe dysphagia (FILS ≤ 6) were excluded, leaving 419 patients with impaired swallowing function. Subsequently, patients transferred to other hospitals due to worsening medical conditions (*n* = 25), those who declined to complete the SNAQ‐JE (*n* = 48), and those unable to complete the SNAQ‐JE (*n* = 37) were excluded. Ultimately, 309 patients were included in the final analysis (mean age 80.3 ± 7.9 years; 57.6% female) (Figure S1).

Detailed patient characteristics are presented in Table 1. Poor appetite was identified in 127 (41.1%) patients. The most common primary diagnosis was stroke (54.0%), followed by musculoskeletal diseases (39.5%) and hospital‐associated deconditioning (6.5%). The prevalence of sarcopenia in the overall cohort was 68.3%. Patients with poor appetite showed a higher prevalence of sarcopenia than those with normal appetite (74.8% vs. 63.7%), although this difference did not reach statistical significance (*p* = 0.053).

**TABLE 1:** Patient characteristics.

	Overall (*n* = 309)	Poor appetite (*n* = 127)	Normal appetite (*n* = 182)	*p*
Age, y, mean (±SD)	80.3 (8.0)	79.8 (8.0)	80.6 (8.0)	0.374
Female sex, *n* (%)	183 (59.2)	79 (62.2)	104 (57.1)	0.439
Primary diagnosis, *n* (%)				0.171
Stroke	167 (54.0)	69 (54.3)	98 (53.8)	
Musculoskeletal disease	122 (39.5)	46 (36.2)	76 (41.8)	
Hospital‐associated deconditioning	20 (6.5)	12 (9.4)	8 (4.4)	
Days from disease onset to admission, d, median [IQR]	24 [17–33]	24 [18–35]	25 [17–32]	0.949
CCI, points, median [IQR]	1 [1–2]	1 [1–2]	1 [1–2]	0.616
BMI, kg/m^2^, mean ± SD	20.4 (3.4)	20.2 (3.3)	20.6 (3.5)	0.272
MUST, points, median [IQR]	2 [1–4]	3 [1–4]	2 [0–3]	< 0.001
SMI, kg/m^2^, mean (±SD)	5.4 (1.2)	5.2 (1.2)	5.6 (1.2)	0.003
HGS, kg, mean (±SD)	17.9 (8.2)	16.8 (8.3)	18.6 (8.2)	0.052
Sarcopenia, *n* (%)	211 (68.3)	95 (74.8)	116 (63.7)	0.053
FILS, points, mean (±SD)	7.7 (0.5)	7.6 (0.5)	7.7 (0.5)	0.11
FIM, points, median [IQR]	76 [58–91]	75 [57–88]	78 [61–93]	0.132
MMSE, points, median [IQR]	24 [19–28]	24 [19–27]	24 [19–28]	0.828
SNAQ‐JE, points, median [IQR]	15 [14–16]	13 [12–14]	16 [15–16]	< 0.001

*Note:* Poor appetite was defined as a score of ≤ 14 on the SNAQ‐JE.

Abbreviations: BMI, Body mass index; CCI, Charlson Comorbidity Index; FILS, Food Intake Level Scale; FIM, Functional Independence Measure; HGS, Handgrip strength; IQR, Interquartile range; MMSE, Mini‐Mental State Examination; MUST, Malnutrition Universal Screening Tool; SD, Standard deviation; SMI, Skeletal muscle mass index; SNAQ‐JE, Simplified Nutritional Appetite Questionnaire for the Japanese elderly.

Comparisons of discharge variables are presented in Table 2. The median hospitalization duration was 50 days [IQR: 37–71], with no significant difference between appetite groups (*p* = 0.206). However, patients with poor appetite had significantly lower FILS scores at discharge compared to those with normal appetite (8.0 ± 0.9 vs. 8.3 ± 0.8, *p* = 0.009). Impaired swallowing function at discharge was also more prevalent in the poor appetite group (78.0% vs. 65.9%, *p* = 0.031). No significant difference was observed in discharge FIM scores (*p* = 0.523).

**TABLE 2:** Comparison of variables at discharge.

	Overall	Poor appetite	Normal appetite	*p*
Length of hospital stay, d, median [IQR]	50 [37–71]	53 [39–76]	50 [35–67]	0.206
FILS, points, mean (±SD)	8.2 (0.9)	8.0 (0.9)	8.3 (0.8)	0.009
FIM, points, median [IQR]	102 [81–114]	99 [81–114]	104 [82–114]	0.523
Presence of impaired swallowing function, *n* (%)	219 (70.9)	99 (78.0)	120 (65.9)	0.031

Abbreviations: FILS, Food Intake Level Scale; FIM, Functional Independence Measure; IQR, Interquartile range; SD, Standard deviation.

**FIGURE 1:**
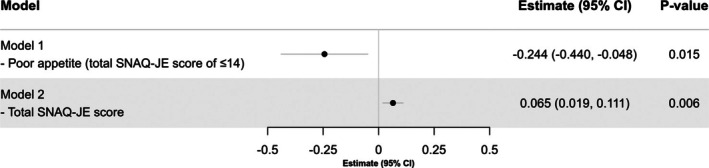
Association between swallowing function at discharge and appetite and SNAQ‐JE score in a multivariable linear regression model. In all models, covariates were age, sex, q disease (cerebrovascular disease, musculoskeletal disorders, and hospital‐associated deconditioning), Charlson Comorbidity Index, days from disease onset to admission, Mulnutrition Universal Screening Tool score, and presence of sarcopenia, Food Intake Level Scale at admission, Functional Independence Measure at admission, and Mini‐Mental State Examination at admission. Abbreviations CI, Confidence interval; SNAQ‐JE, Simplified Nutritional Appetite Questionnaire for the Japanese elderly.

**FIGURE 2:**
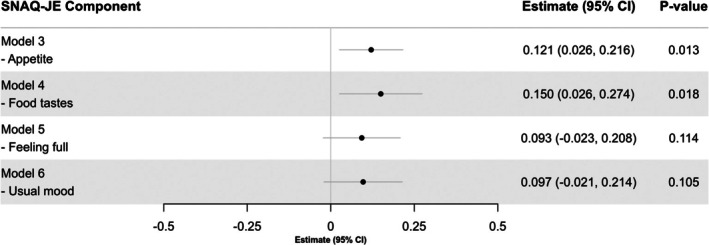
Association between swallowing function at discharge and appetite and SNAQ‐JE subitems in a multivariable linear regression model. In all models, covariates were age, sex, primary diagnosis (cerebrovascular disease, musculoskeletal disorders, and hospital‐associated deconditioning), Charlson Comorbidity Index, days from disease onset to admission, Mulnutrition Universal Screening Tool score, and presence of sarcopenia, Food Intake Level Scale at admission, Functional Independence Measure at admission, and Mini‐Mental State Examination at admission. Abbreviations CI, Confidence interval; SNAQ‐JE, Simplified Nutritional Appetite Questionnaire for the Japanese elderly.

Figure 1 summarizes the results from multivariable linear regression analyses examining the associations between poor appetite and swallowing function at discharge. After adjusting for potential confounders, poor appetite was significantly associated with lower FILS scores at discharge (Estimate: −0.244, 95% CI: −0.440 to −0.048, *p* = 0.015). In the sensitivity analysis using total SNAQ‐JE scores as a continuous variable, better appetite was positively associated with recovery of swallowing function at discharge (Estimate: 0.065, 95% CI: 0.019 to 0.111, *p* = 0.006). Additionally, multivariable linear regression analyses evaluating the associations between individual SNAQ‐JE components and swallowing function at discharge demonstrated that higher scores (indicating better conditions) on the “appetite” (Estimate: 0.121, 95% CI: 0.026 to 0.216, *p* = 0.013) and “food tastes” (Estimate: 0.150, 95% CI: 0.026 to 0.274, *p* = 0.018) were significantly associated with recovery of swallowing function. Conversely, no significant associations were found for “feeling full” (*p* = 0.114) and “usual mood” (*p* = 0.105) items (Figure 2).

**FIGURE 3:**
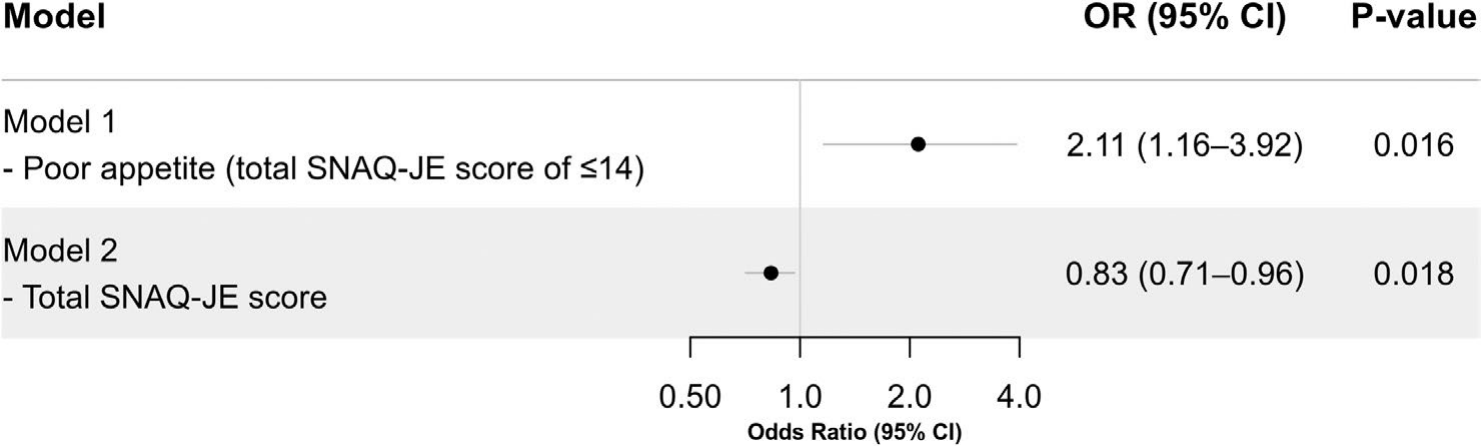
Association between impaired swallowing function at discharge and poor appetite assessed by SNAQ‐JE subitems in a multivariable logistic regression model. In both models, covariates were age, sex, primary diagnosis (cerebrovascular disease, musculoskeletal disorders, and hospital‐associated deconditioning), Charlson Comorbidity Index, days from disease onset to admission, Mulnutrition Universal Screening Tool score, and presence of sarcopenia, Food Intake Level Scale at admission, Functional Independence Measure at admission, and Mini‐Mental State Examination at admission. Abbreviations SNAQ‐JE, Simplified Nutritional Appetite Questionnaire for the Japanese elderly; OR, odds ratio; CI, Confidence interval.

Figure 3 presents the results from multivariable logistic regression analyses, evaluating the association between appetite status and the presence of impaired swallowing function at discharge. After adjusting for potential confounders, poor appetite was significantly associated with an increased likelihood of impaired swallowing function at discharge (odds ratio [OR]: 2.11, 95% confidence interval [CI]: 1.16 to 3.92, *p* = 0.016). In the sensitivity analysis using total SNAQ‐JE scores as a continuous variable, higher scores (indicating better appetite) were significantly associated with a lower likelihood of impaired swallowing function at discharge (OR: 0.83, 95% CI: 0.71–0.96, *p* = 0.018).

Figure S2 presents the exploratory subgroup analyses stratified by primary diagnosis. In the stroke subgroup (*n* = 167), poor appetite was associated with lower FILS scores at discharge (Estimate, −0.280; 95% CI, −0.494 to −0.066), whereas the non‐stroke subgroup (*n* = 142) showed a smaller estimate in the same direction with greater imprecision (Estimate, −0.164; 95% CI, −0.520 to 0.192). The confidence intervals overlapped between subgroups. In the analysis using the continuous SNAQ‐JE score, positive associations with swallowing function were observed in both subgroups (stroke: 0.075; 95% CI, 0.023 to 0.128; non‐stroke: 0.057; 95% CI, −0.026 to 0.140), again with overlapping confidence intervals.

## 4 Discussion

This retrospective cohort study examined the impact of poor appetite on recovery of swallowing function among older adults with impaired swallowing function undergoing post‐acute rehabilitation. By excluding severe dysphagia, we reduced confounding from treatment heterogeneity, cognitive deficits, and physiological barriers, allowing clearer interpretation. Among patients who maintained oral intake, poor appetite at admission was negatively associated with recovery of swallowing function. Furthermore, our results identified that subjective ‘appetite’ and ‘food tastes’ were key components associated with swallowing recovery.

Results from this study align with a previous study suggesting that poor appetite adversely affects overall functional recovery in patients with stroke undergoing rehabilitation [6]. Appetite is closely linked to nutritional status and may influence functional outcomes, given its close association with frailty, sarcopenia, and malnutrition [4, 5, 15]. Reduced energy intake due to poor appetite may contribute to muscle wasting, including oropharyngeal muscles, which are critical for swallowing function. Furthermore, previous studies have reported a positive association between energy intake and improvements in functional ability [7, 16]. Therefore, poor appetite could exacerbate patient vulnerability by limiting energy intake, subsequently impeding swallowing function recovery and overall rehabilitation progress.

A secondary finding of the study was the significant association between specific appetite components and recovery of swallowing function. Among the four components assessed, subjective appetite and food tastes were significantly associated with recovery of swallowing function. Subjective appetite may reflect patient motivation or willingness to eat, while food taste perception is closely linked to sensory pleasure and thus motivation for food intake. Previous studies involving patients with stroke undergoing rehabilitation have reported that reduced motivation can negatively impact behavioral changes and functional recovery [17, 18]. Thus, in this study population, reduced subjective appetite and impaired taste perception may indirectly hinder swallowing function recovery by decreasing motivation for food intake. These findings suggest that the motivational aspects of appetite may serve as important targets for interventions aimed at enhancing rehabilitation outcomes in older adults with impaired swallowing function.

Although statistically significant, the magnitude of the association observed in this study was relatively modest. This may be attributable to our study's specific focus on patients with non‐severe swallowing impairment (FILS 7–8). This population has less capacity for large functional gains compared to those with severe dysphagia. This may have resulted in a ceiling effect on recovery. In addition, exploratory subgroup analyses stratified by primary diagnosis suggested a somewhat larger association in the stroke subgroup. However, the confidence intervals overlapped between subgroups, and no formal tests of interaction were performed. These subgroup findings should be interpreted descriptively, as they may reflect sampling variability rather than true effect modification.

Several limitations of this study should be noted. First, the study was conducted at a single rehabilitation hospital, which may limit the generalizability of other settings. Second, due to the observational nature of this study, causal inferences between appetite and recovery of swallowing function should be interpreted with caution. Unmeasured confounding remains a key limitation. For instance, psychological factors such as depressive symptoms could influence both appetite and swallowing recovery. Furthermore, within the stroke subgroup, neurological factors like lesion location could also act as a common cause for both variables, potentially explaining part of the observed association. Although the SNAQ‐JE includes an item on usual mood, comprehensive psychological assessment was not performed. Third, our analysis did not account for the confounding effects of medications—such as antidepressants, antipsychotics, and sedatives—that are common in this patient population. These drugs can influence oral intake and swallowing through various mechanisms (e.g., appetite modulation, xerostomia), and their frequent use in combination creates a significant potential for confounding interactions. Finally, a significant limitation of our study is the lack of objective swallowing assessments. In this study, swallowing recovery was evaluated solely using the FILS. The FILS cannot objectively measure the underlying physiological swallowing function or identify specific impairments such as pharyngeal residue or laryngeal penetration. The absence of instrumental evaluations, such as a videofluoroscopic swallowing study or fiberoptic endoscopic evaluation of swallowing, therefore restricts a definitive assessment of physiological recovery. Therefore, our findings should be interpreted with caution, as they reflect recovery in functional oral intake rather than directly in the recovery of swallowing physiology.

## 5 Conclusions

Poor appetite was negatively associated with recovery of swallowing function among older patients undergoing post‐acute rehabilitation. Early evaluation of appetite may help identify barriers to recovery of swallowing function, supporting the integration of appetite assessments into routine clinical practice.

## Conflicts of Interest

The authors declare no conflicts of interest.

## Supporting information


**Figure S1:** Flow chart of study. Abbreviations: FILS, Food Intake Level Scale; SNAQ‐JE, Simplified Nutritional Appetite Questionnaire for the Japanese elderly.

**Figure S2:** Exploratory subgroup analyses of the association between appetite and swallowing function, stratified by primary diagnosis (stroke vs. non‐stroke). Forest plots of covariate‐adjusted associations: (a) l poor appetite with FILS at discharge (linear regression); (b) SNAQ‐JE score with FILS at discharge (linear regression); (c) poor appetite with impaired swallowing at discharge (logistic regression); and (d) SNAQ‐JE score with impaired swallowing at discharge (logistic regression). Analyses were stratified by primary diagnosis (stroke vs. non‐stroke; the latter included musculoskeletal disorders and hospital‐associated deconditioning). Models were adjusted for age, sex, Charlson Comorbidity Index, days from disease onset to admission, Mulnutrition Universal Screening Tool score, and presence of sarcopenia, FILS at admission, Functional Independence Measure at admission, and Mini‐Mental State Examination at admission. No formal interaction tests were performed. Estimates are presented as β coefficients (linear regression) or OR (logistic regression) with 95% CI for qualitative comparison. Abbreviations: FILS, Food Intake Level Scale; SNAQ‐JE, Simplified Nutritional Appetite Questionnaire for the Japanese Elderly; CI, confidence interval; OR, odds ratio.

## Data Availability

Research data are not shared.
