# Supplementary figures and images for: Poor Appetite Negatively Affects Recovery of Swallowing Function During Post‐Acute Rehabilitation
Source: Geriatr Gerontol Int. 2025 Sep 29;25(11):1609–15. doi: 10.1111/ggi.70198 (PMC12584940; doi:10.1111/ggi.70198)

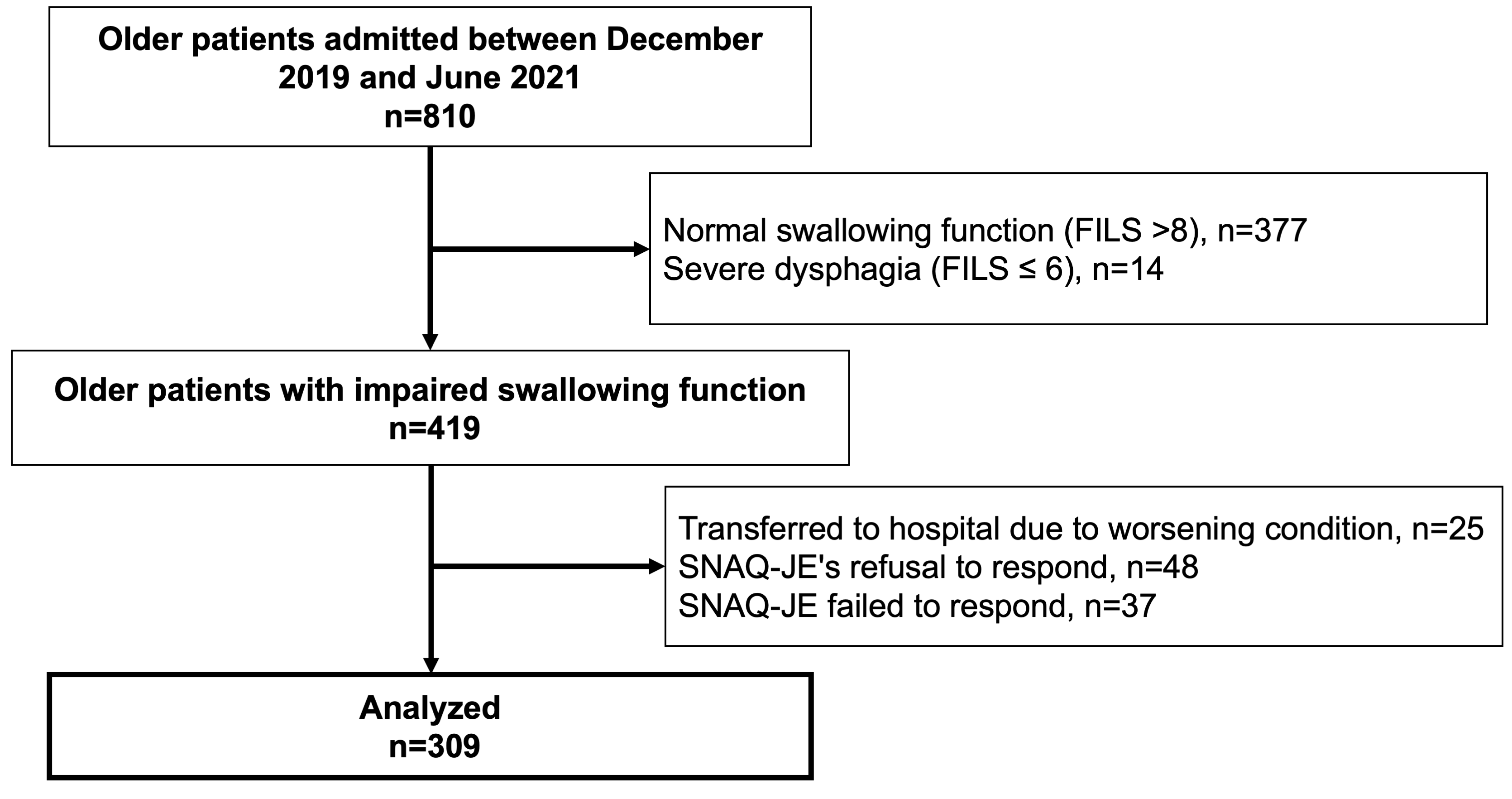

Supplement: Supplementary file 1 — Figure S1: Flow chart of study. Abbreviations: FILS, Food Intake Level Scale; SNAQ‐JE, Simplified Nutritional Appetite Questionnaire for the Japanese elderly. [file GGI-25-1609-s001.tiff]

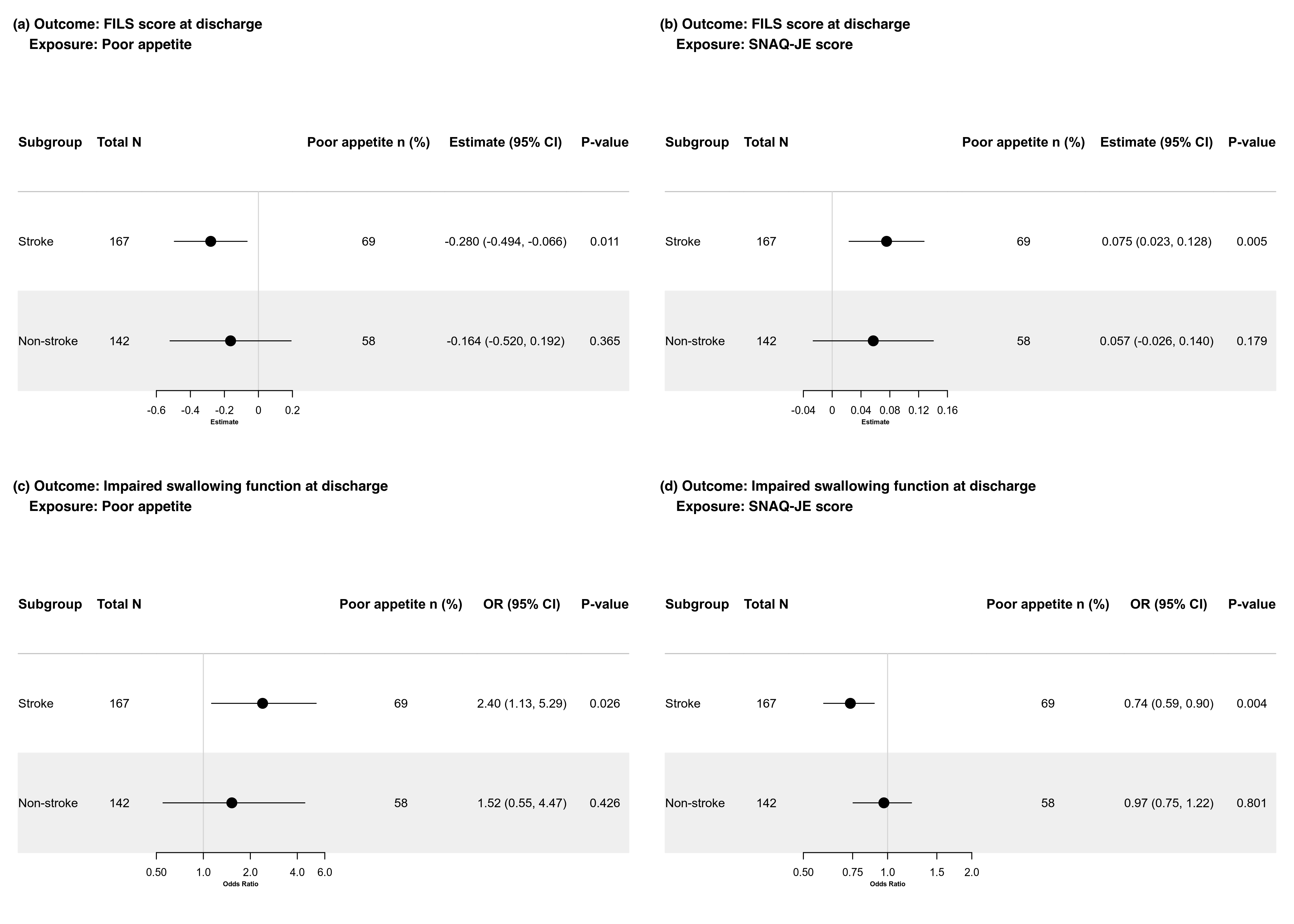

Supplement: Supplementary file 2 — Figure S2: Exploratory subgroup analyses of the association between appetite and swallowing function, stratified by primary diagnosis (stroke vs. non‐stroke). Forest plots of covariate‐adjusted associations: (a) l poor appetite with FILS at discharge (linear regression); (b) SNAQ‐JE score with FILS at discharge (linear regression); (c) poor appetite with impaired swallowing at discharge (logistic regression); and (d) SNAQ‐JE score with impaired swallowing at discharge (logistic regression). Analyses were stratified by primary diagnosis (stroke vs. non‐stroke; the latter included musculoskeletal disorders and hospital‐associated deconditioning). Models were adjusted for age, sex, Charlson Comorbidity Index, days from disease onset to admission, Mulnutrition Universal Screening Tool score, and presence of sarcopenia, FILS at admission, Functional Independence Measure at admission, and Mini‐Mental State Examination at admission. No formal interaction tests were performed. Estimates are presented as β coefficients (linear regression) or OR (logistic regression) with 95% CI for qualitative comparison. Abbreviations: FILS, Food Intake Level Scale; SNAQ‐JE, Simplified Nutritional Appetite Questionnaire for the Japanese Elderly; CI, confidence interval; OR, odds ratio. [file GGI-25-1609-s002.tiff]
